# Improvement of early developmental competence of postovulatory‐aged oocytes using metaphase II spindle injection in mice

**DOI:** 10.1002/rmb2.12335

**Published:** 2020-06-27

**Authors:** Tatsuyuki Ogawa, Hiroko Fukasawa, Shuji Hirata

**Affiliations:** ^1^ Department of Obstetrics and Gynecology Faculty of Medicine University of Yamanashi Chuo Japan

**Keywords:** assisted reproductive technology, infertility treatment, intracytoplasmic sperm injection, metaphase II spindle transfer, oocyte

## Abstract

**Purpose:**

Assisted reproductive technology (ART) is a widely applied fertility treatment. However, the developmental competence of aged oocytes from women of a late reproductive age is seriously reduced and the aged oocytes often fail in fertilization even when ART is used. To resolve this problem, we examined usefulness of a new method “the metaphase II spindle transfer (MESI)” as ART using mouse oocytes.

**Methods:**

This work was composed of two experiments. First, 24 hours after collection, embryos from oocytes (1‐day‐old oocytes, called postovulatory‐aged oocytes), were observed, after intracytoplasmic sperm injection (ICSI), and it was found that they were not able to reach the blastocyst stage. Next, the metaphase II chromosome‐spindle complexes from 1‐day‐old oocytes were injected into cytoplasts from oocytes just collected, using piezo pulses to generate reconstructed oocytes. This procedure was named metaphase II spindle injection (MESI).

**Results:**

After ICSI, embryos from the reconstructed oocytes (32/105), which contained the genes of 1‐day‐old oocytes, were able to develop into the blastocyst stage. The fragmentation rate after ICSI was 28.6%. Thus, the developmental competence of 1‐day‐old oocytes was improved by MESI.

**Conclusions:**

The MESI method has the potential to improve the success rate of infertility treatments for women of a late reproductive age.

## Graphical abstract

The metaphase II chromosome‐spindle complexes from 1‐day‐old oocytes (postovulatory‐aged oocytes 24 hours after collection) were injected into cytoplasts from freshly collected oocytes using piezo pulses to generate reconstructed oocytes (MESI). After ICSI, the embryos from reconstructed oocytes (32/105) could develop into the blastocyst stage. However, the developmental competence of 1‐day‐old oocytes was improved by MESI.

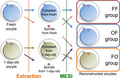

## 1 INTRODUCTION

In 1978, in vitro fertilization was used for the first time leading to the birth of the world's first in vitro fertilized baby, as reported by Edwards and Steptoe.^1^ Subsequently, pregnancy using intracytoplasmic sperm injection (ICSI) was achieved in 1992^2^ and the assisted reproductive technology (ART) became increasingly popular. At present, the birth of one newborn in approximately 16 newborns in Japan is a result of ART.

However, there are difficulties associated with infertility that must be resolved. One difficulty includes embryonic developmental disorders due to the aging of oocytes. It has been considered that the decline in cytoplasmic function of oocytes due to reproductive aging in women is a factor underlying developmental failure, and studies on the rejuvenation of aged oocytes with cytoplasmic replacement have been performed.^3^

**Figure 1:**
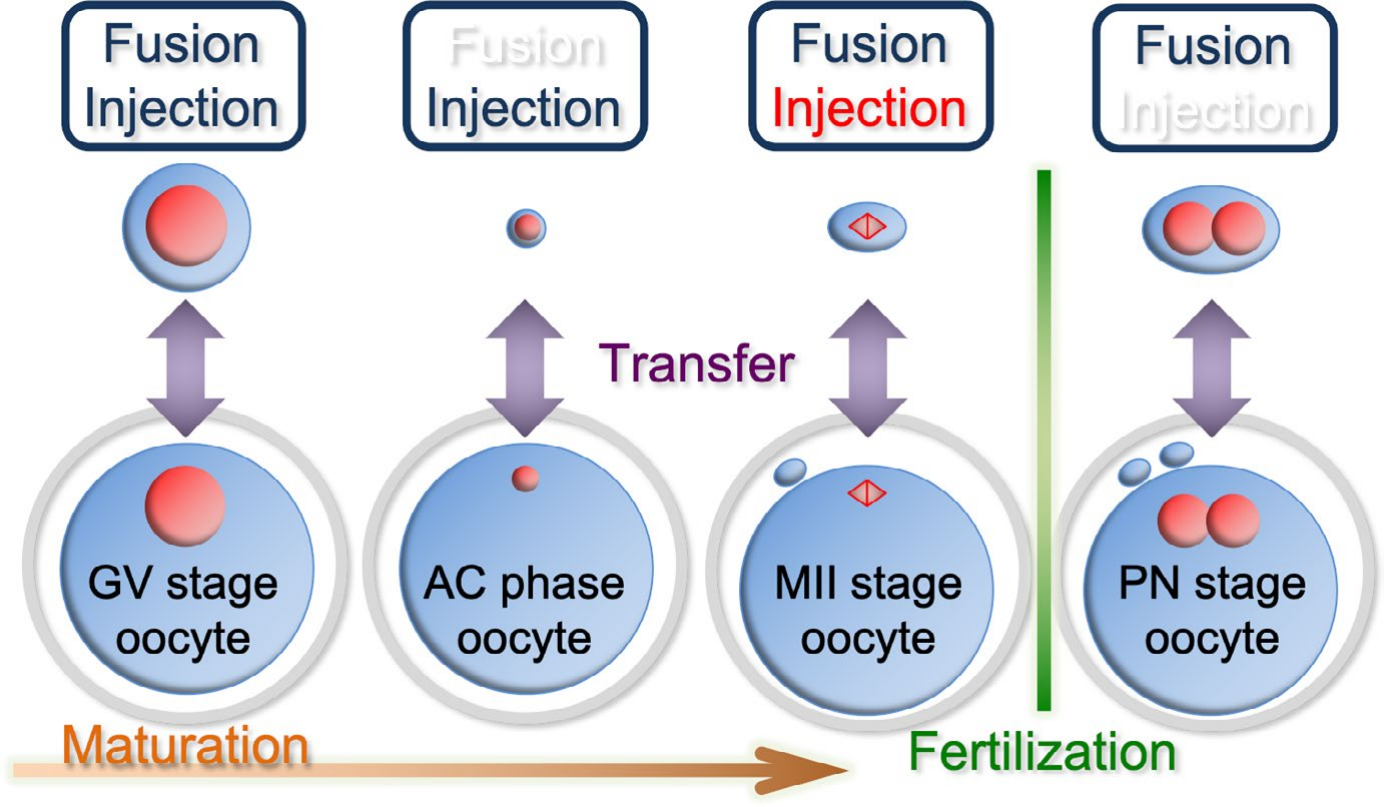
Means of cytoplasmic displacement divided into two main methods: cellular membrane fusion (Fusion) or a direct injection into cytoplasm (Injection). These are administered in each developmental stage of oocytes. In the upper boxes, previously reported methods have been indicated by ultramarine colored letters, new methods have been indicated by white, and the present study has been indicated by red. GV, germinal vesicle; AC, aggregated chromosome; MII, metaphase II; PN, pronuclear

Reported methods of cytoplasmic replacement are broadly grouped into two major categories. Of these, the more widely used method is cellular membrane fusion, which is performed with electrostimulation, inactivated Sendai virus, or polyethylene glycol. Fusion methods are further grouped according to the oocyte stage: germinal vesicle (GV),^4^, ^5^, ^6^, ^7^, ^8^, ^9^ metaphase II (MII),^10^, ^11^, ^12^ or pronuclear^13^, ^14^ (Figure 1). In particular, reconstructed oocytes generated by transferring GVs from oocytes of older patients into enucleated immature oocytes of young patients matured more successfully to meiosis II after an in vitro culture than the nonmanipulated control oocytes.^4^

In the fusion method, the cytoplasm of aged oocytes is carried with nuclear genes into young enucleated oocytes. Reconstructed cytoplasm thus contains numerous mitochondria from the donor oocytes as well, and hence, heteroplasmy of the mitochondrial deoxyribonucleic acid (mtDNA) occurs. Since mtDNA from two different people exist in this scenario, cytoplasmic replacement is not approved in a clinical setting by the United States Food and Drug Administration (FDA). Therefore, the research focus of cytoplasmic replacement has changed from improving the developmental competence of aged oocytes to depressing the level of heteroplasmy.

The degree of mixed mtDNA can be decreased to less than 1% with a cytoplasmic replacement of the human MII oocytes by a cellular membrane fusion (spindle‐chromosomal complex transfer; ST).^15^, ^16^ Moreover, offspring of monkeys generated from reconstructed oocytes by ST were born and grew uneventfully.^17^ In 2016, the FDA accepted only male fertilized eggs, and in the next year, the first boy derived from oocyte spindle transfer, to prevent mitochondrial disease, was reported.^18^ It is increasingly likely that a cytoplasmic replacement will be applied to humans.^19^

The other method of a cytoplasmic replacement, which was reported recently, is the injection of karyoplasts into the cytoplast directly.^20^, ^21^, ^22^ This is a promising method in which a mixed mtDNA used for the reconstruction of oocytes is decreased as compared to that in a cellular membrane fusion. With a piezo‐driven system, GV injection has been established.^20^ Alternatively, an aggregated chromosome (AC) transfer has been demonstrated^21^; the AC phase lasts for 1‐4 hours between a GV breakdown and the metaphase I stage in human oocytes.^22^ In an AC transfer, fine injection pipettes with a tip internal diameter of 5‐6 µm are used. This method is excellent as it only slightly damages oocytes. However, an AC transfer cannot be performed with murine oocytes that do not have an AC phase.^22^

The current study attempted to decrease the mixed mtDNA in reconstructed oocytes via the injection of the MII chromosome‐spindle complex, which was extracted using a micropipette, and the cytoplasm around the spindle was cleared away mechanically (we named this metaphase II spindle injection; MESI). Moreover, going back to the original idea of a cytoplasmic replacement, this study aimed to improve the competence of aged oocytes.

In this study, murine MII oocytes cultured in vitro without fertilization after collection (called postovulatory‐aged oocytes^23^) were treated as a model of aged oocytes. Since oocytes after collection are altered by temperature, medium, or environmental factors such as reactive oxygen species,^24^, ^25^, ^26^, ^27^, ^28^ it has been suggested that the window for optimal fertilization is 8‐12 hours after ovulation.^23^ Originally, aged oocytes were collected from women of an impaired reproductive function due to aging (called reproductive aging women^29^). These two types of aged oocytes have similar alterations such as MII aberrations, spontaneous activation, cellular fragmentation, the initiation of an apoptotic pathway,^30^ chromosome aneuploidy,^31^ and reduced mitochondrial function.

In postovulatory‐aged oocytes, the level of intracytoplasmic reactive oxygen species becomes elevated, and the resultant oxidative stress affects the intracellular Ca^2+^ regulation and impairs the embryonic development after fertilization.^32^ Meanwhile, oocytes from women experiencing reproductive aging exhibit distinct morphological changes and a significant decrease in the number of mitochondria. Thus, reproductive competence is reduced.^33^, ^34^ In addition, in murine oocytes from females undergoing reproductive aging, the relative telomerase activity (RTA) is significantly lower and the relative telomere length (RTL) is remarkably shorter as compared to those in young oocytes. In contrast, postovulatory aging has no significant effect on the RTA and RTL of oocytes.^35^

As described, there are some similarities and differences between postovulatory‐aged murine oocytes and oocytes from women undergoing a reproductive aging. However, these are not congruent with each other because of the differences in the mechanisms of aging. Based on the major commonality of depressed developmental competence, this study treated postovulatory‐aged oocytes as a model of aged oocytes.

Previously, the cytoplasmic replacement methods did not involve direct injection of MII oocytes (MESI) karyoplasts into the cytoplast. The present study, using reconstructed oocytes with the new method “MESI,” examined the possibility of improving the competence of aged oocytes and the relationship between the functional decline of the spindle and cytoplasm, and developmental disorders of aged oocytes.

## 2 MATERIALS AND METHODS

### 2.1 Animals

Female and male B6D2F1 mice (C57BL/6N × DBA/2N, registered as Jcl), 8‐10 weeks of age, were obtained from SLC (Shizuoka, Japan). Mice were handled according to the Guidelines of the Center for Life Science Research, University of Yamanashi. Mice were given food and water ad libitum and were maintained under a 14‐hour light/10‐hour dark cycle.

### 2.2 Preparation of oocytes

Female B6D2F1 mice were administered 5 IU of a pregnant mare's serum gonadotropin intraperitoneally. After 48 hours, they were given 5 IU of the human chorionic gonadotropin in the same manner. Cumulus‐oocyte complexes were collected from oviducts 16 hours after the human chorionic gonadotropin injection. Cumulus‐oocyte complexes were denuded to the cumulus cells in a modified HEPES‐buffered CZB medium^36^ containing 0.1% (w/v) hyaluronidase and were sorted from the MII oocytes morphologically. The oocytes were then placed in the KSOM medium (Specialty Media, Phillipsburg, NJ, USA) containing amino acids, glucose, and 1 mg/mL bovine serum albumin at 37°C with a humidified mixture of 5% (v/v) CO_2_ and 95% (v/v) air.

Oocytes were divided into two groups. One group included fresh oocytes that were collected within 4 hours. The other contained 1‐day‐old postovulatory oocytes maintained in the incubator for 24 hours after collection.

### 2.3 Preparation of sperm

Semen obtained from the cauda epididymis of male B6D2F1 mice was placed into the KSOM medium in the incubator. Thirty minutes later, sperm in the supernatant fluid was mixed with 10% (w/v) polyvinylpyrrolidone (*M*r 360,000, FUJIFILM Wako Pure Chemical Corporation, Osaka, Japan).

### 2.4 ICSI

Before ICSI, sperm tails were removed using piezo pulses (Prime Tech, Ibaraki, Japan) with an injection pipette that had a tip internal diameter of 5‐6 µm. A sperm head was injected into the ooplasm using piezo pulses^36^ in the modified HEPES‐buffered CZB medium. Considering damage to the cellular membrane, oocytes were maintained at room temperature (24‐25°C) for 15 minutes after ICSI and then placed in the incubator.

### 2.5 MESI

**Figure 2:**
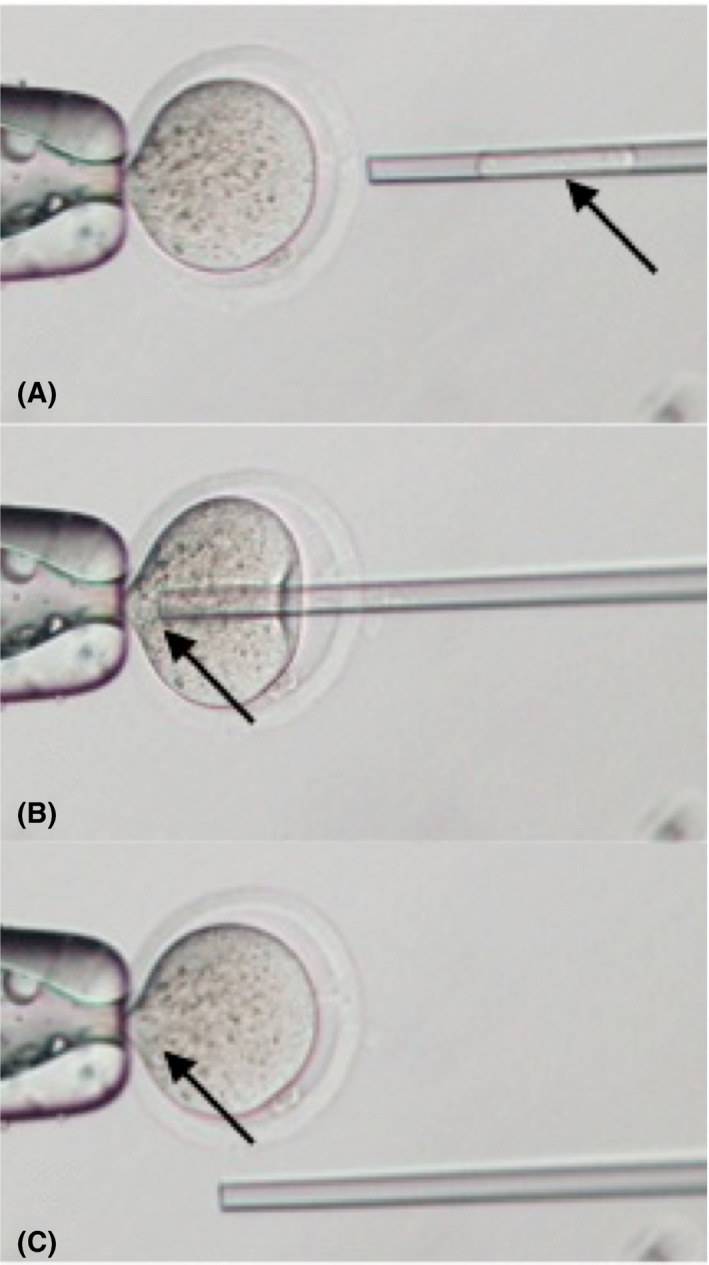
Pictures indicating the method of a metaphase II spindle injection (MESI). The held oocyte in the left is enucleated, and a spindle from another oocyte is contained in the right micropipette and the spindle is minimalized by a gentle pipetting (A). After the cellular membrane is broken with piezo pulses, the spindle is injected into the enucleated ooplasm (B). The injected spindle is near the holding pipette, and the oocyte has been reconstructed (C)

MII oocytes were treated with 5 µg/mL cytochalasin B in the HEPES‐buffered CZB before enucleation. The MII chromosome‐spindle complex was removed using a micropipette with a tip internal diameter of 7‐8 µm^37^ and divided into spindles with little cytoplasm (karyoplast) and cytoplast samples. By pipetting once or twice gently with a spindle injection pipette (having a tip internal diameter of 6‐7 µm), the cytoplasm around the spindle was cleared away mechanically (we called “purified”). This manipulation was performed because the spindle is harder than the cytoplasm and thus more transmissive. The purified karyoplast was prepared by breaking its plasma membrane in the spindle injection pipette using piezo pulses, followed by injection into other enucleated oocytes to generate reconstructed oocytes (Figure 2). Considering damage to the spindle and cellular membrane, reconstructed oocytes were stored in an incubator at 37°C for 2 hours before ICSI.

#### 2.5.1 Experiment 1

To evaluate the developmental competence of 1‐day‐old oocytes, embryos derived from fresh and 1‐day‐old oocytes after ICSI were observed for development into the blastocyst stage.

#### 2.5.2 Experiment 2

**Figure 3:**
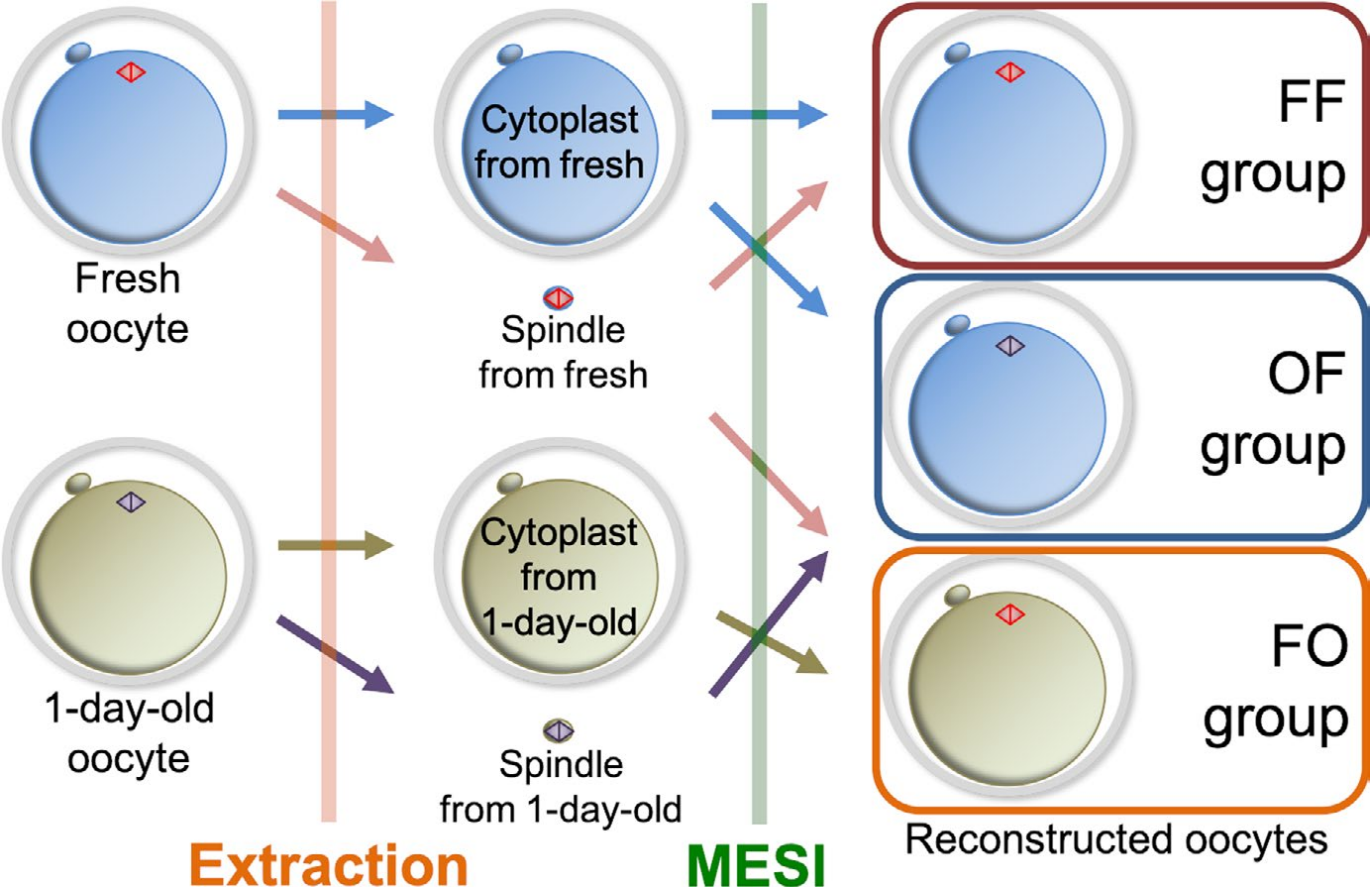
Combinations of the three groups in the metaphase II spindle injection (MESI) of experiment 2

**Figure 4:**
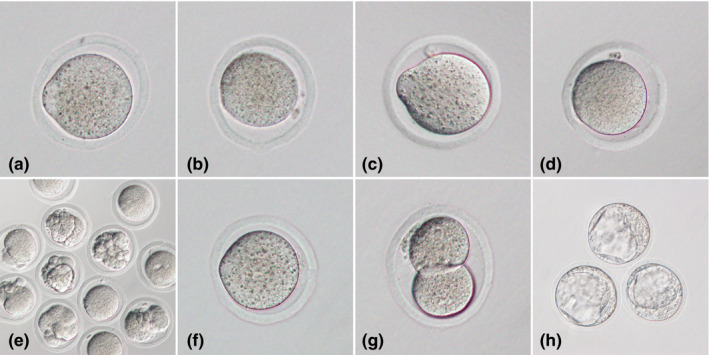
Representative images of oocytes or embryos in this study. A, Fresh oocyte. B, 1‐day‐old oocyte. C, Reconstructed oocyte from FF group (cytoplast from fresh oocyte and spindle from another fresh oocyte). D, Reconstructed oocyte from FO group (cytoplast from 1‐day‐old oocyte and spindle from fresh oocyte). E, Most of the reconstructed oocytes from FO group had fragmentation. F, Reconstructed oocyte from OF group (cytoplast from fresh oocyte and spindle from 1‐day‐old oocyte). G, Embryo in the two‐cell stage from OF group. H, Embryos in the blastocyst stage from OF group

In an effort to examine functions of the spindle and cytoplasm in early development, three groups of reconstructed oocytes were generated by MESI. One group was called the FF (fresh, fresh) group, which was composed of spindle and cytoplast from fresh oocytes. Another was called FO (fresh, 1‐day‐old) group, which consisted of the spindle from fresh oocytes and the cytoplast from 1‐day‐old oocytes. The third was called the OF (1‐day‐old, fresh) group, which was made up of spindle from 1‐day‐old oocytes and cytoplast from fresh oocytes (Figure 3). Embryos derived from the three groups of reconstructed oocytes after ICSI were observed for development to the blastocyst stage (Figure 4). In addition, the quantitative test of mtDNA contained in the purified karyoplast used for MESI was performed using real‐time PCR.

### 2.6 Statistics

Data were compared using the Chi‐squared tests, or the Fisher's exact test when n < 5. The significance was set at *P* < .05. For comparisons between groups, the Bonferroni's correction was used.

## 3 RESULTS

### 3.1 Experiment 1

The rates of survival, fragmentation, and development into the blastocyst stage for fresh and 1‐day‐old oocytes have been shown in Table 1. All surviving 1‐day‐old oocytes arrested in the one‐ or two‐cell stage, with or without fragmentation after ICSI, and never developed to blastocysts. Thus, the rate of blastocyst embryo development of 1‐day‐old oocytes was significantly lower than that of fresh oocytes (0% vs 76.7%, respectively; *P* < .01). In addition, the rate of fragmentation in fresh oocytes was 6.3% and that in 1‐day‐old oocytes was 61.8% (*P* < .01).

**Table 1:** Results of experiment 1, intracytoplasmic sperm injection (ICSI) for fresh and 1‐day‐old oocytes

	N	No. surviving after ICSI (% of N)	No. of fragments (% of survived)	No. of PN (% of survived)	No. of. embryos developing to	
					2‐cell stage (% of PN)	Blastocyst stage (% of PN)
Fresh	141	128 (90.8)	8 (6.3)	120 (93.8)	114 (95.0)	92 (76.7)
1‐day‐old	138	131 (94.9)	81 (61.8)	50 (38.2)	29 (58.0)	0 (0)
*P* value		.246	<.01	<.01	<.01	<.01

Abbreviations: ICSI, intracytoplasmic sperm injection; PN, pronuclear.

### 3.2 Experiment 2

The results of survival and development in the three groups of reconstructed oocytes after MESI and ICSI have been indicated in Table 2 and Figure 4. The rates of blastocyst embryo development were significantly different between the FF, FO, and OF groups (50.3 vs 0% vs 30.5%, respectively; *P* < .01). All surviving oocytes of the FO group after MESI arrested in the one‐ or two‐cell stage, with or without fragmentation, and no embryo developed to the blastocyst stage. The rates of fragmentation in the FO group were 56.8% after MESI and 54.0% after ICSI. These were significantly higher than the rates in the other groups (*P* < .01). Comparing the FF and OF groups, the rates of fragmentation after MESI were not significantly different between the groups (2.2% vs 5.8%, respectively; *P* = .055); however, after ICSI, they were significantly different (8.3% vs 28.6%, respectively; *P* < .01).

**Table 2:** Results of experiment 2, metaphase II spindle transfer (MESI), and intracytoplasmic sperm injection (ICSI), for the three groups of FF, FO, and OF

	N	No. surviving after MESI (% of N)	No. of fragments after MESI (%)	No. surviving after ICSI (%)	No. of fragments after ICSI (%)	No. of PN (%)	No. of. embryos developing to	
							2‐cell stage (% of PN)	Blastocyst stage (% of PN)
FF	334	231 (69.2)	5 (2.2)^a^	156 (67.5)	13 (8.3)^c^	143 (91.7)	133 (93.0)	72 (50.3)^e^
FO	393	155 (39.4)	88 (56.8)^b^	63 (40.6)	34 (54.0)^d^	29 (46.0)	12 (41.4)	0 (0)^f^
OF	402	223 (55.5)	13 (5.8)^a^	147 (65.9)	42 (28.6)^c^	105 (71.4)	87 (82.9)	32 (30.5)^e^

Abbreviations: FF, cytoplast from fresh oocyte and spindle from another fresh oocyte; FO, cytoplast from 1‐day‐old oocyte and spindle from fresh oocyte; ICSI, intracytoplasmic sperm injection; MESI, metaphase II spindle injection; OF, cytoplast from fresh oocyte and spindle from 1‐day‐old oocyte; PN, pronuclear.

a vs. b: *P* < .01, c vs. d: *P* < .01, e vs. f: *P* < .01

In the quantitative test of mtDNA, when the mtDNA contained in a whole oocyte was 100%, the mtDNA contained in the purified karyoplast used for MESI was only 0.36% (Table 3).

**Table 3:** Results of experiment 2, the quantitative test of mitochondrial deoxyribonucleic acid (mtDNA) contained in the purified karyoplast by real‐time PCR

	N	The quantity of mtDNA (mean ± SD)
A whole oocyte	3	100 ± 16.0
Purified karyoplast	3	0.357 ± 0.428

Abbreviation: mtDNA, mitochondrial deoxyribonucleic acid.

## 4 DISCUSSION

### 4.1 MESI as a new method

As reconstructed oocytes of the FF group developed into the blastocyst stage, cytoplasmic replacement by MESI was achieved. Using MESI, it was possible to make the cytoplasm evidently smaller, with the clearance of unnecessary cytoplasm around the spindle by pipetting, as compared to that with a cellular membrane fusion. As the cytoplasm around the spindle contains many mitochondria, its reduction is beneficial. As a result, we were able to reduce the amount of mtDNA contained in the purified karyoplast to 0.36% of a whole oocyte. This was not inferior to the previous reports.^15^, ^16^ Therefore, one advantage of MESI was that the amount of mixed mtDNA delivered to the reconstructed oocytes could be decreased.

Previously, the FDA did not allow the application of cytoplasmic replacement in a clinical setting because of the unclear risk associated with the artificial phenomenon of the coexistence of mtDNA from two different individuals. Owing to the effect of a decreasing carriage of the mtDNA, MESI may move cytoplasmic replacement closer to a clinical application.

ST for MII oocytes^15^, ^16^ has received a substantial attention recently because excluding abnormal mitochondria by ST may help to prevent the onset of mitochondrial diseases.^38^, ^39^, ^40^ In the United Kingdom, there are more families with mitochondrial disorders than in other countries. Thus, in February 2015, the United Kingdom Parliament approved regulations to permit the use of maternal spindle and pronuclear transfer.^41^ Moreover, it appears that MESI can produce equivalent or better outcomes than ST with a cytoplasmic fusion. Whereas the use of genetic material from three separate sources (maternal nuclear DNA, donor mtDNA, and paternal sperm) poses unique ethical questions,^42^, ^43^, ^44^ MESI requires further research to enhance its safety and effectiveness.

### 4.2 Fragmentation rates

The rates of fragmentation were not significantly different among 1‐day‐old oocytes after ICSI (61.8%), the FO group after MESI (56.8%), and the FO group after ICSI (54.0%). The common link among these three groups is that they possess cytoplasm from 1‐day‐old oocytes. As the mechanism of fragmentation remains to be clarified, manipulation of the cytoplasm from 1‐day‐old oocytes may directly cause fragmentation or induce it indirectly via activation. Furthermore, the fragmentation rate of the OF group after ICSI was significantly higher than that of the FF group (28.6% vs 8.3%, respectively; *P* < .01). This suggested that the spindle might have a suppressive function on fragmentation and that the function of the spindle from 1‐day‐old oocytes was depressed.

### 4.3 Blastocyst development rates

Based on the results of experiment 1, the difference in early developmental competence between fresh and 1‐day‐old oocytes was evident, and experiment 2 was conducted to examine the cause. Though no 1‐day‐old oocytes after ICSI developed into the blastocyst stage, 30.5% of the reconstructed oocytes of the OF group reached this stage. Further, no reconstructed oocytes of the FO group reached the blastocyst stage. From these results, it was clear that, whether composed of the spindle from fresh or 1‐day‐old oocytes, reconstructed oocytes could develop into the blastocyst stage if the cytoplasm was from fresh oocytes. Moreover, it was evident that oocytes could not develop to the blastocyst stage if the cytoplasm was from 1‐day‐old oocytes.

Additionally, the blastocyst rates of the FF and OF groups, which both had cytoplasm from fresh oocytes, differed significantly (50.3% vs 30.5%, respectively; *P* < .01). From this, it was considered that both the spindle and the cytoplasmic function affected early developmental competence and that the cytoplasm had a greater effect.

However, the blastocyst rate of the FF group was lower than the rate (87.3%) reported by a previous study using ST,^45^ probably because in MESI, the oocyte membrane was broken, decrease the durableness. In addition, MESI requires higher technical expertise than that required by ST. This problem can, however, be solved by improving the manipulation techniques and the embryo culture environment in the future.

The present study with MESI demonstrated that developmental disorders of 1‐day‐old oocytes were caused principally by a decrease in the cytoplasmic function. Moreover, with fresh ooplasm, MESI was able to improve the depressed developmental competence of 1‐day‐old oocytes. The successful improvement of developmental competence of postovulatory‐aged oocytes (1‐day‐old in this study) suggested the possibility that the competence of oocytes from elderly women undergoing reproductive aging could be improved. In addition, MESI could reduce the amount of mtDNA carried with the transferred spindle. However, to apply MESI in a clinical setting, more advanced research on mtDNA heteroplasmy will be needed.

## DISCLOSURES

*Conflict of interest*: Tatsuyuki Ogawa, Hiroko Fukasawa, and Shuji Hirata declare that they have no conflict of interest. *Human and animal rights*: This article does not contain any studies with human subjects performed by the any of authors. All institutional and national guidelines for the care and use of laboratory animals were followed. The experimental protocol of this study was approved by the Animal Care and Use Committee and Ethics Committee, University of Yamanashi.
